# Cost-Effectiveness of Automated Digital Microscopy for Diagnosis of Active Tuberculosis

**DOI:** 10.1371/journal.pone.0157554

**Published:** 2016-06-20

**Authors:** Swati Jha, Nazir Ismail, David Clark, James J. Lewis, Shaheed Omar, Andries Dreyer, Violet Chihota, Gavin Churchyard, David W. Dowdy

**Affiliations:** 1 Department of Epidemiology, Johns Hopkins Bloomberg School of Public Health, Baltimore, United States of America; 2 Centre for Tuberculosis National Institute for Communicable Diseases, Sandringham, South Africa; 3 Department of Medical Microbiology, University of Pretoria, Pretoria, South Africa; 4 Aurum Institute, Johannesburg, South Africa; 5 MRC Tropical Epidemiology Group, Department of Infectious Disease Epidemiology, London School of Hygiene and Tropical Medicine, London, United Kingdom; 6 School of Public Health, University of the Witswatersrand, Johannesburg, South Africa; Public Health Research Institute at RBHS, UNITED STATES

## Abstract

**Background:**

Automated digital microscopy has the potential to improve the diagnosis of tuberculosis (TB), particularly in settings where molecular testing is too expensive to perform routinely. The cost-effectiveness of TB diagnostic algorithms using automated digital microscopy remains uncertain.

**Methods:**

Using data from a demonstration study of an automated digital microscopy system (TBDx, Applied Visual Systems, Inc.), we performed an economic evaluation of TB diagnosis in South Africa from the health system perspective. The primary outcome was the incremental cost per new TB diagnosis made. We considered costs and effectiveness of different algorithms for automated digital microscopy, including as a stand-alone test and with confirmation of positive results with Xpert MTB/RIF (‘Xpert’, Cepheid, Inc.). Results were compared against both manual microscopy and universal Xpert testing.

**Results:**

In settings willing to pay $2000 per incremental TB diagnosis, universal Xpert was the preferred strategy. However, where resources were not sufficient to support universal Xpert, and a testing volume of at least 30 specimens per day could be ensured, automated digital microscopy with Xpert confirmation of low-positive results could facilitate the diagnosis of 79–84% of all Xpert-positive TB cases, at 50–60% of the total cost. The cost-effectiveness of this strategy was $1280 per incremental TB diagnosis (95% uncertainty range, UR: $340-$3440) in the base case, but improved under conditions likely reflective of many settings in sub-Saharan Africa: $677 per diagnosis (95% UR: $450-$935) when sensitivity of manual smear microscopy was lowered to 0.5, and $956 per diagnosis (95% UR: $40-$2910) when the prevalence of multidrug-resistant TB was lowered to 1%.

**Conclusions:**

Although universal Xpert testing is the preferred algorithm for TB diagnosis when resources are sufficient, automated digital microscopy can identify the majority of cases and halve the cost of diagnosis and treatment when resources are more scarce and multidrug-resistant TB is not common.

## Background

Every year, an estimated 3.6 million individuals develop active tuberculosis (TB) yet are not notified to public health authorities[1]. Improved diagnostic testing for TB is likely to be critical for reaching this “missing 3 million”[2, 3]. Current testing algorithms largely depend either on sputum smear microscopy—a test with variable sensitivity that depends on operator skill and such variables as microscopist time constraints[4]–or Xpert MTB/RIF (Cepheid, Inc.; Sunnyvale, CA, USA), a molecular test with higher sensitivity and the ability to detect rifampin resistance[5, 6], but that can cost over ten times more per test than sputum smear[3, 7]. For settings in which high-quality sputum smear microscopy cannot be performed, but performing Xpert MTB/RIF on all patients with symptoms of TB may be very expensive (including settings of systematic screening for TB), automated digital microscopy offers an attractive alternative[8]. Automated visualization algorithms can facilitate high-quality sputum smear microscopy that is not user-dependent in interpretation; an example of an automated microscopy system that was recently validated is the TBDx system (Applied Visual Sciences, Leesburg, VA, USA)[9]. This system uses patented software and a high-specification camera to identify acid fast bacilli, thereby reducing subjectivity in results and potentially improving performance. In a prospective single-center study at the National Tuberculosis Reference Laboratory in Johannesburg, South Africa, TBDx was shown to have 0.78 sensitivity for culture-proven TB, and when positive results were confirmed by Xpert MTB/RIF, the specificity of the algorithm was estimated at 0.998[9]. However, whether TBDx or a similar automated microscopy system could be cost-effective in high-burden settings remains uncertain. Thus, we used data from this study to perform an economic evaluation of automated digital microscopy for active TB in a representative African reference lab setting.

## Methods

### Overview

Like most automated microscopy systems, TBDx provides results at different levels of certainty; in the case of TBDx, as reported in the published demonstration study [9], there are four relevant result levels: negative (no acid-fast bacilli [AFB] detected), “scanty 1” (<1 AFB per 300 high-power fields), “low positive” (2–9 AFB per 300 high-power fields), and “high positive” (≥10 AFB per 300 high-power fields). We used data on these TBDx results, alone and in combination with Xpert MTB/RIF, as performed in 1009 South African adults with clinical suspicion of TB, to construct a model of the effectiveness of such testing in a hypothetical population of 10,000 people having characteristics of the source population. In addition to diagnostic consequences, we estimated the costs of TB testing and treatment from a health system perspective. The original study was approved by the Faculty of Health Sciences Research Ethics Committee at the University of Pretoria; no additional ethical approval was required for this non-human subjects analysis.

We evaluated the costs and effectiveness of a series of alternative algorithms: (1) sputum smear microscopy alone; (2) TBDx automated microscopy alone; (3) TBDx automated microscopy, with confirmation of low positive results by Xpert MTB/RIF; (4) TBDx automated microscopy, with confirmation of all positive results by Xpert MTB/RIF; and (5) Xpert MTB/RIF performed on all specimens. For each algorithm including automated microscopy, we considered scenarios in which “scanty 1” results were treated as either negative or low positive. Under each scenario, we projected the total number of patients with culture-confirmed TB who would be treated, the number of false-positive diagnoses that would be made, and the total costs from a health system perspective. For purposes of appropriate comparison (to scenarios in which treatment of drug-resistant TB would otherwise not be considered), we also considered options in which drug susceptibility testing was performed for all individuals before initiation of TB treatment. We also considered three alternative scenarios of treatment volume: a “high-volume” scenario in which 100 individuals were evaluated per day, a “moderate-volume” scenario in which 30 individuals were evaluated per day and a “low-volume” scenario in which 10 individuals were evaluated per day. Our primary outcome was the incremental cost per incremental true-positive diagnosis made, relative to sputum smear microscopy alone as the reference scenario.

### Diagnostic costs

All costs were valued in 2015 US dollars, with conversion from South African rand into US dollars using the historical exchange rate and inflation to year 2015 using the South African gross domestic product (GDP) deflator[10]. Unit costs were developed for sputum smear, TBDx automated microscopy, and Xpert MTB/RIF using estimates from the literature in an “ingredients” approach as shown in Table 1. Overheads (including building space) and utilities were estimated from other published evaluations of sputum smear microscopy and Xpert MTB/RIF in South Africa, conservatively assuming that allocations for TBDx-enhanced microscopy would be similar to Xpert MTB/RIF (and thus higher than for microscopy) and also including an annual cost for quality assurance and training, the allocation of which to each type of diagnostic modality was assumed to be $400[11–13].

**Table 1:** Unit Costs of Diagnostic Tests for Tuberculosis in South Africa (2015 US$).

Cost components	Sputum Smear Microscopy			Automated Digital Microscopy			Xpert MTB/RIF		
	*100 samples/d*	*30 samples/d*	*10 samples/d*	*100 samples/d*	*30 samples/d*	*10 samples/d*	*100 samples/d*	*30 samples/d*	*10 samples/d*
**Utilities and overheads**	0.08	0.14	0.31	0.10	0.17	0.40	0.14	0.26	0.59
Equipment*	0.01	0.02	0.06	0.65	1.57	4.70	1.92	2.33	3.67
**Staff**	1.37	1.37	1.37	0.47	0.47	0.47	1.32	1.32	1.32
**Licensing**	0.00	0.00	0.00	2.00	2.10	6.25	0.00	0.00	0.00
Consumables**	0.12	0.12	0.12	0.12	0.12	0.12	11.48	11.48	11.48
**Total**	**$1.59**	**$1.65**	**$2.07**	**$3.35**	**$4.43**	**$12.14**	**$14.45**	**$15.39**	**$16.94**

*inclusive of shipping and installation cost, annual warranty, repair and maintenance cost;

** Inclusive of shipping and distribution cost

Costs of capital equipment (e.g., microscopes and Xpert systems) were also taken from the literature and annualized using a 3% annual discount rate and an expected useful life of five years (Xpert) or ten years (microscope and camera)[14, 15]. Costs of required hardware and software licensing for TBDx were valued in consultation with experts from Applied Visual Systems, Inc.–who provided estimates of cost without any input as to the use of those estimates in the economic model. These estimates included: (a) a licensing fee of $2 per slide in a high-volume setting or $15,000 per year in low and moderate-volume setting; (b) combined equipment costs of slide loader-fitted microscope and camera of $27,000 for a low and moderate-throughput loader and $47,000 for a high-throughput loader; (c) $3,600 in installation and shipping costs; (d) $1,000 for a computer and printer (also applied to the Xpert MTB/RIF); and (e) annual maintenance and warranty at 10% of total equipment costs (also applied to Xpert and fluorescence microscopes).

Personnel costs were estimated assuming an hourly wage of $5.13 for a general laboratory technician and $6.83 for a skilled microscopist[11, 12, 16]. We assumed that all procedures related to Xpert specimen preparation, loading, and results reporting would take 15 minutes per test (run on-demand), versus five minutes per slide for sputum smear (where individual smears can be batched), plus an additional seven minutes per slide for manual reading. Consumables were estimated at $0.10 for sputum smear (including TBDx) and $9.98 for Xpert MTB/RIF (the current cost of an Xpert cartridge)[13, 15, 17, 18], with procurement and shipping costs estimated at 10% of the unit price.

### Treatment costs

We estimated the cost of TB treatment according to the cost of first-line and second-line drugs as well as necessary outpatient follow-up. We did not attempt to estimate downstream consequences after initiation of treatment; thus, all people diagnosed with TB were assigned a treatment cost equivalent to drugs and outpatient visits for six months (if drug-susceptible) or twenty months (if multidrug-resistant, MDR), and all people not diagnosed with TB were treated as false-negative or true-negative, without attempting to capture future attempts at diagnosis or empiric treatment. We assumed that culture-based drug susceptibility testing would be performed prior to any initiation of MDR-TB therapy, and we included the costs thereof. We assumed a high proportion of MDR-TB (5% of all cases), as might be reflective of testing performed at a national referral center. Costs of diagnosis with standard sputum smear microscopy and Xpert MTB/RIF, as well as other component costs of automated microscopy (e.g., staff costs, overhead costs, DST) were estimated from the literature[12, 13, 16, 19, 20]. A full listing of model parameters is given in Table 2.

**Table 2:** Model Parameters.

Parameter	Value	Sensitivity range	Reference
Proportion of patients with active TB	0.108	0.05–0.2	[1, 9]
Proportion of TB that is resistant to rifampin	0.09	0.02–0.2	[1, 19]
Proportion of TB that is multi-drug resistant	0.03	0.01–0.07	
Sensitivity for culture-confirmed TB:			[9]
Sputum smear microscopy	0.68	0.4–0.68	
TBDx (any positive)	0.80	0.7–0.9	
TBDx (>1 AFB/300 fields)	0.73	0.6–0.85	
TBDx (high positive)	0.62	0.55–0.7	
Xpert MTB/RIF (TBDx +)	0.97	0.95–1.0	
Xpert MTB/RIF (all TB)	0.91	0.8–1.0	
Specificity:			[9]
Sputum smear microscopy	0.992	0.98–1.0	
TBDx (any positive)	0.79	0.7–0.9	
TBDx (>1 AFB/300 fields)	0.96	0.92–0.98	
TBDx (high positive)	0.998	0.99–1.0	
Xpert MTB/RIF (TBDx +)	0.97	0.95–1.0	
Xpert MTB/RIF (all TB)	0.990	0.98–1.0	
Cost to treat one patient			[3, 13, 16, 19, 20, 29]
Drug-susceptible TB	$506	$300-$700	
Drug-resistant TB	$3660	$2000-$10,000	
Daily capacity			Assumption
Fluorescence microscope	50		
High-throughput TBDx	>100		
Xpert MTB/RIF(4-module system)	16		[15]

### Sensitivity analyses

We performed one-way sensitivity analysis on all model parameters across reasonable ranges as shown in Table 2. In addition, we also performed scenario analyses for high- and low-volume settings (as above) and at different assumed levels of MDR-TB prevalence. In addition, we performed a probabilistic uncertainty analysis in which all parameters were simultaneously varied over the ranges shown in Table 2 (and by +/-25% of base value for each cost component not shown in Table 2). Each parameter range was defined as a beta distribution with an alpha (shape) parameter of 4. The results of this analysis are reported as 95% uncertainty ranges (95% UR’s), bounded by the 2.5^th^ and 97.5^th^ percentiles of the resulting simulations.

## Results

### Impact of volume on cost of automated microscopy

In a low-volume setting processing 10 specimens per day, the unit cost of automated digital microscopy was nearly that of Xpert MTB/RIF (Table 1), due to the relatively high costs of both equipment and annual software licensing for TBDx. By contrast, in both moderate (30 specimens/day) and high-volume (100 specimens/day) settings, the per-test cost of automated microscopy was about one-third that of Xpert MTB/RIF. Given the relatively low likelihood that automated microscopy would be performed instead of Xpert MTB/RIF if the unit costs of testing were similar, we focused subsequent analyses on the high-volume setting.

### Costs and consequences of automated digital microscopy

**Fig 1:**
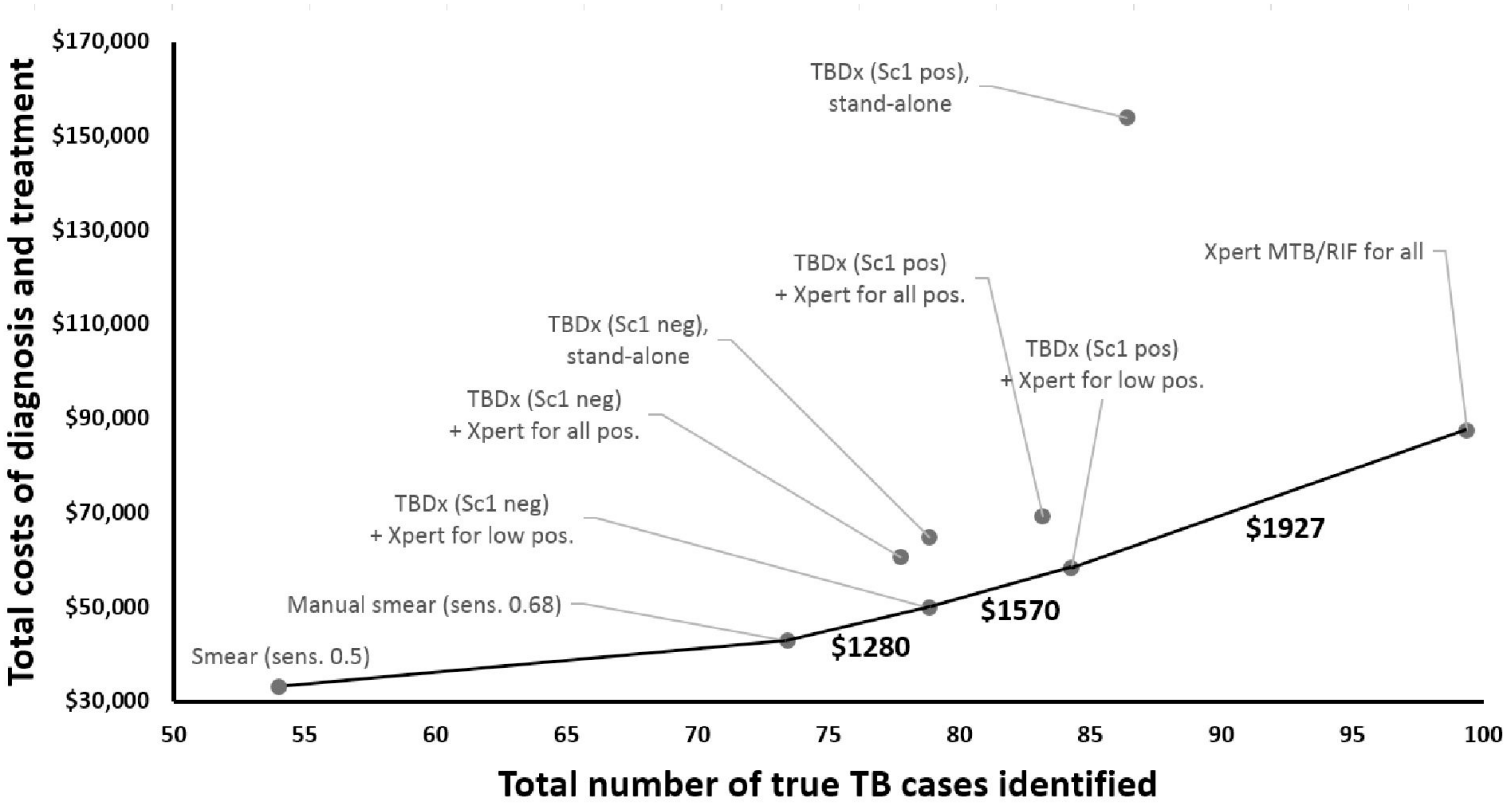
Cost-Effectiveness Frontier. The number of true-positive microbiological diagnoses for each algorithm is shown on the x-axis and the corresponding costs on the y-axis, such that the slope of any line is the incremental cost-effectiveness ratio between two algorithms. Algorithms appearing further to the right are more effective, and those appearing higher on the y-axis are more expensive. The frontier of cost-effective options is shown as a solid dark line, with incremental cost-effectiveness ratios (in units of cost per true-positive TB treatment) shown for each comparison along this frontier. In a setting of constrained resources, automated digital microscopy with low-positive results confirmed by Xpert MTB/RIF would be the first selected strategy beyond sputum smear microscopy alone, followed by Xpert MTB/RIF for all specimens, where resources are sufficient. If one incremental microbiological diagnosis could avert as few as 0.2 disability-adjusted life years, all strategies along the cost-effectiveness frontier would be cost-effective, at a willingness-to-pay threshold equal to South Africa’s per-capita annual gross national income (GNI). Sc1 = “scanty 1” result; pos = positive; neg = negative; sens. = sensitivity.

Table 3 shows the costs and consequences of various testing algorithms for active TB that incorporate automated digital microscopy. Using automated microscopy as a standalone test (i.e., no confirmation of positive results by Xpert MTB/RIF) resulted in more false-positive diagnoses than incremental true-positive diagnoses. However, if automated microscopy were used as a triage test, with low-positive results confirmed by Xpert MTB/RIF and high-positive results proceeding to treatment, 79–84% of all individuals with Xpert-positive TB could be diagnosed (true TB diagnoses made), at 50–60% of the cost (total diagnostic cost plus total treatment cost). This strategy had a favorable incremental cost-effectiveness ratio of $1,280 per TB diagnosis made (95% uncertainty range, UR: $340-$3440), relative to manual sputum smear microscopy with sensitivity of 0.68 (versus 0.73 for TBDx). If manual sputum smear had a sensitivity of 0.5 rather than 0.68 (as observed in the TBDx demonstration study), this automated microscopy strategy would cost $677 per incremental TB diagnosis (95% UR: $450-$935), relative to manual smear. If the prevalence of MDR-TB was lowered to 1% of all samples, the cost of this algorithm fell to $956 per diagnosis (95% UR: $40-$2910). The corresponding cost-effectiveness frontier (Fig 1) demonstrates that this strategy would be preferred where resources are insufficient for universal Xpert MTB/RIF, or the willingness to pay for an incremental TB diagnosis falls between $1,280 ($677 if sensitivity of manual smear is 0.5) and $1,927.

**Table 3:** Cost-Effectiveness of Different TB Diagnostic Algorithms Performed on 1000 South African Adults in a High-Volume Setting with 5% Prevalence of MDR TB.

Algorithm	Diagnostic costs		Treatment costs		TB treatments (of 108 with true TB)		False-positive treatments	Incremental cost per true TB diagnosis (relative to manual smear)	
	*Total*	*Incr*.	*Total*	*Incr*.	*Total*	*Incr*.			
**Sputum smear (manual)**									
Sensitivity 0.68	$1,590	REF	$41,300	REF	73.5	REF	8	REF	-
Sensitivity 0.5	$1,590	-	$31,400	-	54	-	8	-	REF
**Automated microscopy**									
*Scanty 1 = low positive*									
Microscopy only (stand-alone)	$3,350	$1,760	$150,600	$109,400	86.4	12.9	211	$8,570	$3,730
Xpert to confirm low positive	$6,370	$4,780	$51,900	$10,600	84.2	10.8	12	$1,430	$835
Xpert to confirm any positive	$7,550	$5,960	$61,600	$20,400	83.1	9.7	11	$2,710	$1,240
*Scanty 1 = negative*									
Microscopy only (stand-alone)	$3,350	$1,760	$61,400	$20,100	78.8	5.4	42	$4,050	$1,280
Xpert to confirm low positive	$4,090	$2,500	$45,700	$4,420	78.9	5.4	7	$1,280	$675
Xpert to confirm any positive	$5,270	$3,680	$55,400	$14,100	77.8	4.3	6	$4,130	$1,160
**Xpert MTB/RIF for all**	$14,700	$13,200	$72,600	$31,400	99.4	25.9	10	$1,720	$1,200

### Sensitivity and scenario analyses

**Fig 2:**
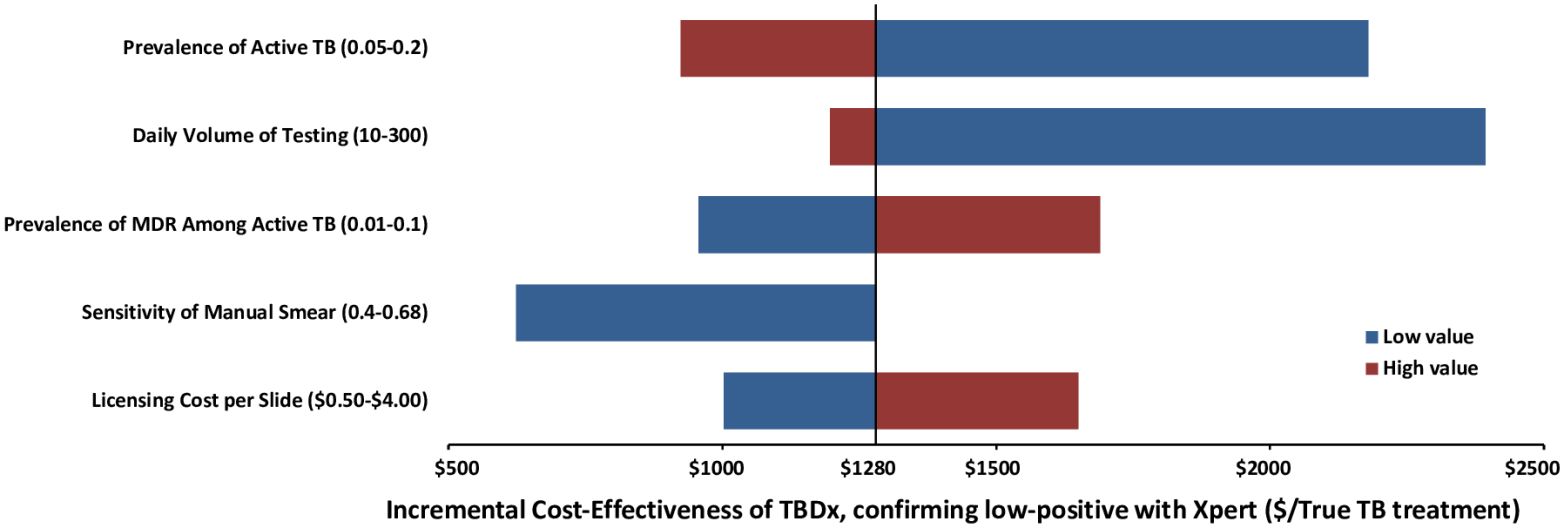
One-Way Sensitivity Analysis. Primary drivers of cost-effectiveness in one-way sensitivity analysis. All parameters in Tables 1 and 2 were varied; only those parameters that resulted in a change of +/-$200 in the incremental cost-effectiveness ratio are shown. Blue bars correspond to the incremental cost-effectiveness of automated microscopy by TBDx relative to manual smear microscopy, at the low value of the specified parameter range. Red bars correspond to the incremental cost-effectiveness at the high value of that range, holding all other parameter values constant.

Assuming that both the specificity of manual sputum smear microscopy and the volume of testing is high, three additional parameters were strong drivers of the incremental cost-effectiveness of automated digital microscopy (Fig 2): the prevalence of TB, the prevalence of MDR-TB, and the sensitivity of manual microscopy. In settings where the sensitivity of manual microscopy is poor, the incremental cost-effectiveness of TBDx with Xpert confirmation of all positive results began to approach that of universal Xpert (Table 3). This strategy allows for 78–83% of all Xpert-positive TB to be diagnosed, with all drug-resistant TB also treated. Under most situations tested in sensitivity analysis, the algorithm that provided the most TB diagnoses per dollar spent (incremental to manual microscopy) was TBDx, with Xpert confirmation of low-positive results, and counting “Scanty 1” results as negative.

## Discussion

This economic evaluation provides insight as to the potential role of automated digital microscopy in the diagnosis of adult pulmonary tuberculosis. Specifically, in settings where universal Xpert MTB/RIF is affordable, and health systems are willing to pay at least $1927 per incremental TB diagnosis made, universal Xpert is generally preferred. If an incremental microbiologically-confirmed TB diagnosis can lead to one year of additional life (or disability-adjusted life year averted), this willingness-to-pay threshold would be considered highly cost-effective by traditional criteria in most high-burden countries[21, 22]. This finding is consistent with prior economic evaluations suggesting that Xpert is likely to be highly cost-effective relative to sputum smear microscopy where the level of empiric TB diagnosis is low[7, 20]. However, in many settings, universal Xpert MTB/RIF is not an affordable option[13], in part owing to recurring maintenance and calibration costs. In these cases, and when specimens can be processed at moderate-to-high volume, automated digital microscopy (with confirmation of positive results by Xpert) can improve the yield of manual microscopy, and at an incremental cost-effectiveness ratio that is favorable relative to universal Xpert. The strategy that appears to optimize cost-effectiveness in most settings is Xpert confirmation of low-positive results only, taking high-positive results as a direct indication for treatment.

Prior analyses have evaluated the potential cost-effectiveness of hypothetical “triage” tests for active TB, suggesting that such tests could be cost-effective under certain conditions[23]. This analysis is among the first to evaluate a novel diagnostic test for TB that could be used for purposes of triage. Such tests are designed to provide the majority of the benefit of using a more sensitive confirmatory test (e.g., Xpert MTB/RIF), but at a reduced cost—and ideally a more favorable cost-effectiveness ratio, such that it is preferable to perform the triage test on the full population rather than the confirmatory test on part of it. This economic evaluation demonstrates precisely this result, specifically that automated digital microscopy can reduce the overall costs of diagnostic testing substantially, and be performed at a cost-effectiveness ratio that is generally favorable to that of universal Xpert.

As shown in our sensitivity analysis, automated microscopy is particularly attractive in settings where manual microscopy cannot be performed with consistent quality. For TBDx as a specific example of automated microscopy, the preferred algorithm is one in which high-positive results lead directly to treatment, low-positive results are confirmed with Xpert (or other more accurate confirmatory test), and inconclusive results (i.e., 1 AFB per 300 fields) are treated as negative. Unless the cost of licensing and equipment can be brought down in the future, TBDx is likely to be too expensive on a per-test basis to scale up in low-volume settings that do not perform substantially more than 10 sputum evaluations for TB each day.

Two elements of automated digital microscopy performance bear mention in this analysis. Automated digital microscopy was performed on concentrated sputum specimens, which may increase the sensitivity of both manual and automated microscopy[24], though perhaps less so in those infected with HIV[25]. Customized slides and marking of the inoculation area for camera guidance were also critical to test performance; though not major contributors to cost, these required elements may limit the ability to perform automated digital microscopy in some settings. Furthermore, while slides are still manually prepared, the automated algorithm does not assess sputum quality.

As with any modeling analysis, this evaluation has certain limitations. Parameter values were drawn from the literature and, in some cases, required assumptions based on expert opinion. Nevertheless, our results were robust to most sensitivity analyses, and particularly surrounding those parameter values that were most uncertain. We did not attempt to incorporate downstream effectiveness measures, including the health benefits of making TB diagnoses nor the costs and effectiveness of HIV therapy, which is a major driver of the economics of TB diagnosis in HIV-endemic settings[26]. Our ability to compare automated microscopy to health interventions other than TB diagnostic assays is therefore limited. Nevertheless, we are able to demonstrate that the cost-effectiveness of automated microscopy is likely similar (if not superior) to that of Xpert MTB/RIF, which has been modeled in such fashion[7]. We limited this analysis to the costs and consequences of microbiological diagnosis; in reality, ancillary diagnostic testing (e.g., with chest X-ray) and empiric treatment may have major impact on patient outcomes[27, 28]. Our results should therefore not be interpreted as reflective of the entire process of TB diagnosis and treatment, but rather only of the costs and consequences of diagnosis and treatment based on microbiological confirmation.

In summary, this economic evaluation suggests that automated digital microscopy can serve as a cost-effective alternative to Xpert MTB/RIF when specimens can be processed at high volume and universal Xpert is unaffordable. The algorithm most likely to be cost-effective is one in which high-positive results on automated microscopy result in referral for treatment, low-positive results are confirmed with Xpert, and inconclusive results are treated as negative. Further studies should evaluate the effectiveness (ideally with linkage to empiric costs) of automated digital microscopy in a variety of real-world settings. As the armamentarium of diagnostic options for TB continues to expand, it is important to optimize the use of each test in such a way that constrained resources are put to their best use. By reducing the costs of Xpert MTB/RIF testing while still providing the majority of the benefits (in terms of TB diagnoses), automated digital microscopy has the potential to fill an important niche in the TB diagnostic landscape.
